# Effects of a person-centred telephone-support in patients with chronic obstructive pulmonary disease and/or chronic heart failure – A randomized controlled trial

**DOI:** 10.1371/journal.pone.0203031

**Published:** 2018-08-31

**Authors:** Andreas Fors, Elin Blanck, Lilas Ali, Ann Ekberg-Jansson, Michael Fu, Irma Lindström Kjellberg, Åsa Mäkitalo, Karl Swedberg, Charles Taft, Inger Ekman

**Affiliations:** 1 Institute of Health and Care Sciences, Sahlgrenska Academy, University of Gothenburg, Gothenburg, Sweden; 2 Centre for Person-Centred Care (GPCC), University of Gothenburg, Gothenburg, Sweden; 3 Närhälsan Research and Development Primary Health Care, Region Västra Götaland, Gothenburg, Sweden; 4 Department of Respiratory medicine and Allergology, Institute of Medicine, Sahlgrenska Academy, University of Gothenburg, Gothenburg, Sweden; 5 Research and Development department, Region Halland, Halmstad, Sweden; 6 Department of Molecular and Clinical Medicine, Sahlgrenska Academy, University of Gothenburg, Gothenburg, Sweden; 7 Department of Education, Communication and Learning, University of Gothenburg, Gothenburg, Sweden; 8 National Heart and Lung Institute, Imperial College, London, United Kingdom; Universite de Bretagne Occidentale, FRANCE

## Abstract

**Purpose:**

To evaluate the effects of person-centred support via telephone in two chronically ill patient groups, chronic obstructive pulmonary disease (COPD) and/or chronic heart failure (CHF).

**Method:**

221 patients ≥ 50 years with COPD and/or CHF were randomized to usual care vs. usual care plus a person-centred telephone-support intervention and followed for six months. Patients in the intervention group were telephoned by a registered nurse initially to co-create a person-centred health plan with the patient and subsequently to discuss and evaluate the plan. The primary outcome measure was a composite score comprising General Self-Efficacy (GSE), re-hospitalization and death. Patients were classified as deteriorated if GSE had decreased by ≥ 5 points, or if they had been re-admitted to hospital for unscheduled reasons related to COPD and/or CHF or if they had died.

**Results:**

At six-month follow-up no difference in the composite score was found between the two study groups (57.6%, n = 68 vs. 46.6%, n = 48; OR = 1.6, 95% CI: 0.9–2.7; P = 0.102) in the intention-to-treat analysis (n = 221); however, significantly more patients in the control group showed a clinically important decrease in GSE (≥ 5 units) (22.9%, n = 27 vs. 9.7%, n = 10; OR = 2.8, 95% CI: 1.3–6.0; P = 0.011). There were 49 clinical events (14 deaths, 35 re-admissions) in the control group and 41 in the intervention group (9 deaths, 32 re-admissions). Per-protocol analysis (n = 202) of the composite score showed that more patients deteriorated in the control group than in the intervention group (57.6%, n = 68 vs. 42.9%, n = 36; OR = 1.8, 95% CI 1.0–3.2; P = 0.039).

**Conclusion:**

Person-centred support via telephone mitigates worsening self-efficacy without increasing the risk of clinical events in chronically ill patients with CHF and/or COPD. This indicates that a patient-healthcare professional partnership may be established without the need for face-to-face consultations, even in vulnerable patient groups.

**Trial registration:**

ISRCTN.com ISRCTN55562827.

## Introduction

Person-centred care (PCC) constitutes a shift away from a one-size-fits-all model of care based on diagnoses to an approach based on ethical principles where a contractual agreement is made involving the patient as an active partner in the care and decision-making process [1, 2]. In the PCC framework developed by the University of Gothenburg Centre for Person-Centred Care (GPCC), patients and healthcare professionals jointly develop a health plan based on the patient´s illness history and medical status in which the patient’s own capabilities and social support resources are identified and documented along with potential barriers to achieve goals [1]. Our point of departure is that health care needs to target patients’ beliefs in their ability to perform desired activities rather than simply attempting to convince them of the value of certain activities [3]. A crucial concept in this regard is self-efficacy, which is defined as a person’s belief in his/her own ability to successfully execute behaviours necessary to produce desired outcomes [4]. Accordingly, a central goal in the GPCC approach is to enable patients’ self-efficacy by stressing the importance of knowing the patient as a person with capabilities and resources to perform activities and achieve desired goals [1].

The effectiveness of PCC as a tool to improve health care has been demonstrated in several controlled trials in different conditions, settings and contexts. For example, in chronic conditions such as chronic heart failure (CHF), PCC has been shown to reduce length of hospital stay [5]; reduce uncertainty about the disease [6] and treatment and improve the efficiency of the discharge process [7]; lower healthcare costs [8, 9]; reduce re-hospitalization rates and improve health-related quality of life [10]; and improve adherence to medications [11]. A study evaluating the effects of PCC along the chain of health care in patients with acute coronary syndromes (ACS) showed a three-fold higher chance [12] (even higher when complemented with an eHealth tool [13]), of improved general self-efficacy in combination with return to work or previous activity level without increasing the risk of cardiovascular events or death. These effects were sustained for up to two years [14] and were particularly pregnant in patients with low socioeconomic status [15]. PCC has also been shown to improve confidence in managing symptoms [16].

Long term health conditions are one of the major challenges facing healthcare systems worldwide [17] and this study focuses on two of them: CHF and chronic obstructive pulmonary disease (COPD). Both of these conditions are associated with high morbidity, high mortality and severe impacts on activities of daily living [18, 19]. In developed countries, the prevalence of CHF is approximately 1–2% in the adult population and ≥ 10% among persons 70 years and older [19]. The global prevalence of COPD is difficult to estimate due to different calculation methods and underdiagnoses, but is estimated to almost 200 million cases worldwide [18]. CHF and COPD often present together and have similar symptomology making it difficult for patients and clinicians to differentiate which condition is responsible for the worsened situation [20, 21]. Common to patients who are diagnosed with CHF and/or COPD is low quality of life with symptoms such as fatigue, shortness of breath and anxiety, which often lead to repeated hospitalizations or outpatient or primary care visits [18, 19, 22]. These patients are also often burdened by uncertainty about their illness and, for patients with COPD, even stigma [6, 23, 24]. Although pharmacological therapy has improved outcome markedly over the last 15–20 years, improved management is needed to optimize care [25].

In order to better address present and future challenges in health care, telehealth has been suggested as a safe option to promote self-management of chronic conditions [26]. In patients with CHF, telehealth has been shown to reduce all-cause mortality and heart failure hospitalizations although these findings are not consistent. For patients with COPD, the impact of telehealth on all-cause mortality has shown no significant differences versus usual care and improved health outcomes are rarely reported [26]. Moreover, in patients with CHF and COPD telehealth has been shown to induce a more active self-management role by patients and families [27]. There are few existing telehealth intervention studies that fully address PCC [28]. We therefore performed a study with the aim to evaluate the effects of a person-centred telephone support in patients with CHF and/or COPD.

## 2. Method

### 2.1 Study design

We performed a randomized, open, parallel-group, controlled intervention study which assessed three and six month outcomes of person-centred telephone support added to usual care versus usual care alone. Randomization was based on a computer-generated list, which stratified for age ≥ 75 and diagnosis (COPD, CHF or COPD and CHF). The Ethical Review Board at the University of Gothenburg approved the study (DNr 687–14) and the investigation conforms to the principles outlined in the Declaration of Helsinki. The study was reported to the ICRCTN-registry in January 2015, thereafter inclusion of participants to the trial was started. Because it was not assigned in the registry until March 2015 the study was classified as retrospectively registered.

### 2.2 Setting and participants

Patients admitted with worsening of CHF and/or COPD at one hospital site within the Sahlgrenska University Hospital in Gothenburg, Sweden were pre-screened from medical records and consecutively enrolled between January 2015 and November 2016. Patients were considered eligible if they were ≥ 50 years, owned a telephone with an active subscription and hospitalized due to worsening CHF and/or COPD. Exclusion criteria were: no registered address within the Västra Götaland region, severe hearing impairment, cognitive impairment, current alcohol or drug abuse, survival expectancy less than one year, or participating in a conflicting study. All patients willing to participate were included in the study during their hospital stay once their condition had stabilized sufficiently. Patients received oral and written information about the study and, after giving written consent to participate, were randomized by registered nurses (RNs) to either the intervention group or the control group. The first follow-up was at three months and the second at six months.

### 2.3 Control group

Patients in the control care group received usual care and were managed as outlined in existing treatment guidelines at study start [19, 29].

### 2.4 Intervention group

In addition to usual care, the patients in the intervention group received a telephone call one to four weeks after discharge. The date and time for the call was agreed upon while the patient was still in hospital. The calls were made by one of four RNs who had received extensive training in person-centred communication and a two day dedicated education about the medical conditions (CHF and COPD). During the whole study period the RNs met regularly every other week with a group of specialists in the areas of person-centredness, communication and pedagogics in order to continuously develop their person-centred communication skills (e.g. listening, open-ended questions, reflections and summaries), documentation and their understanding of the philosophical underpinnings of PCC. In addition, as a part of their training, the RNs reviewed some of each other´s telephone calls and documentation.

During their conversations with the patients, the RNs followed the GPCC approach [1, 12] to build a partnership with the patients. They listened to the patients’ narratives and asked questions in order to identify and deepen their understanding of the patients’ capabilities, resources and potential for self-care. The RNs made efforts to identify patients’ wishes, potentials and discussed problem areas such as dilemmas on how to take prescribed medicines and sleeping problems. The patient and the RNs together formulated attainable goals during the 6 month long study period. After the calls, a brief summary of the conversation as well as goals agreed upon were documented in a health plan which was sent by mail to the patients. The plan also specified how and when the patient and the RN would have further contact during the remaining study period, but it was possible for patients in the intervention group to contact a RN weekdays during office hours. The health plan was evaluated and updated when needed in subsequent telephone conversations between the patient and the RN.

### 2.5 Endpoints

The primary endpoint was a composite score of changes [30] in general self-efficacy (GSE) [31], re-hospitalization and death. Each patient was classified as either deteriorated, improved or unchanged at 6 months as follows:

- Deteriorated: if GSE had decreased by ≥ 5 units OR re-admitted to hospital for unscheduled reasons related to COPD and/or CHF OR had died;
- Improved: if GSE had increased by ≥ 5 units AND the patient had not been hospitalized for unscheduled reasons related to COPD and/or CHF AND not died.
- Unchanged: neither deteriorated nor improved according to the above criteria.

Components of the composite outcome were also analyzed separately.

### 2.6 General self-efficacy scale

The General Self-Efficacy Scale (GSE scale) is a 10-item self-assessment scale designed to assess the strength of person’s beliefs that he/she is capable of dealing effectively with task requirements in a wide range of contexts [31], e.g. dealing efficiently with unexpected events, handling unforeseen situations, and finding solutions to problems. The respondents are asked to rate each item on a 4-point scale (1 = not at all true, 2 = barely true, 3 = moderately true, 4 = exactly true) and ratings are summed to a total score ranging from 10–40, where higher scores indicate higher levels of general self-efficacy. A validated Swedish version of the GSE scale [32] was used in this study. In a previous study, an increase of ≥ 5 points in the GSE scale was considered a threshold for minimal clinically important change [12]. The GSE scale was completed by patients in hospital at baseline and at three and six months was completed at home and returned by post.

### 2.7 Sample size

To achieve 80% power based on an alfa-error of 0.05 for the proportion of patients who deteriorate, compared to those improved or were unchanged, was assumed to decrease from 40% to 20%, the number of participants in each group (comparison and intervention) needed to comprise 91 patients. We aimed to include at least 110 patients in each group to have some margin for withdrawals.

### 2.8 Statistical analysis

Descriptive statistics were used to characterize the study groups. Between-group differences in baseline characteristics were tested using Fisher´s exact test. Logistic regression was used to calculate odds ratios with 95% confidence intervals for an improved composite score. Mann-Whitney U test was used to analyse between-group differences in changes in GSE scores. Last observation carried forward and baseline observation carried forward was used to handle missing GSE data (22.2% at three months and 28.1% at 6 months) and sensitivity analyses were performed to assess robustness. All statistical tests were two-sided with a significance level of P ≤ 0.05 and were performed using IBM SPSS Statistics version 25. The primary analysis was performed according to intention-to-treat principles (ITT analysis). In addition, per-protocol analysis (PP analysis) was performed including intervention patients who had had at least one follow-up of the first contact by telephone, a protocol-specified requirement for fully implemented intervention.

## 3. Results

**Fig 1:**
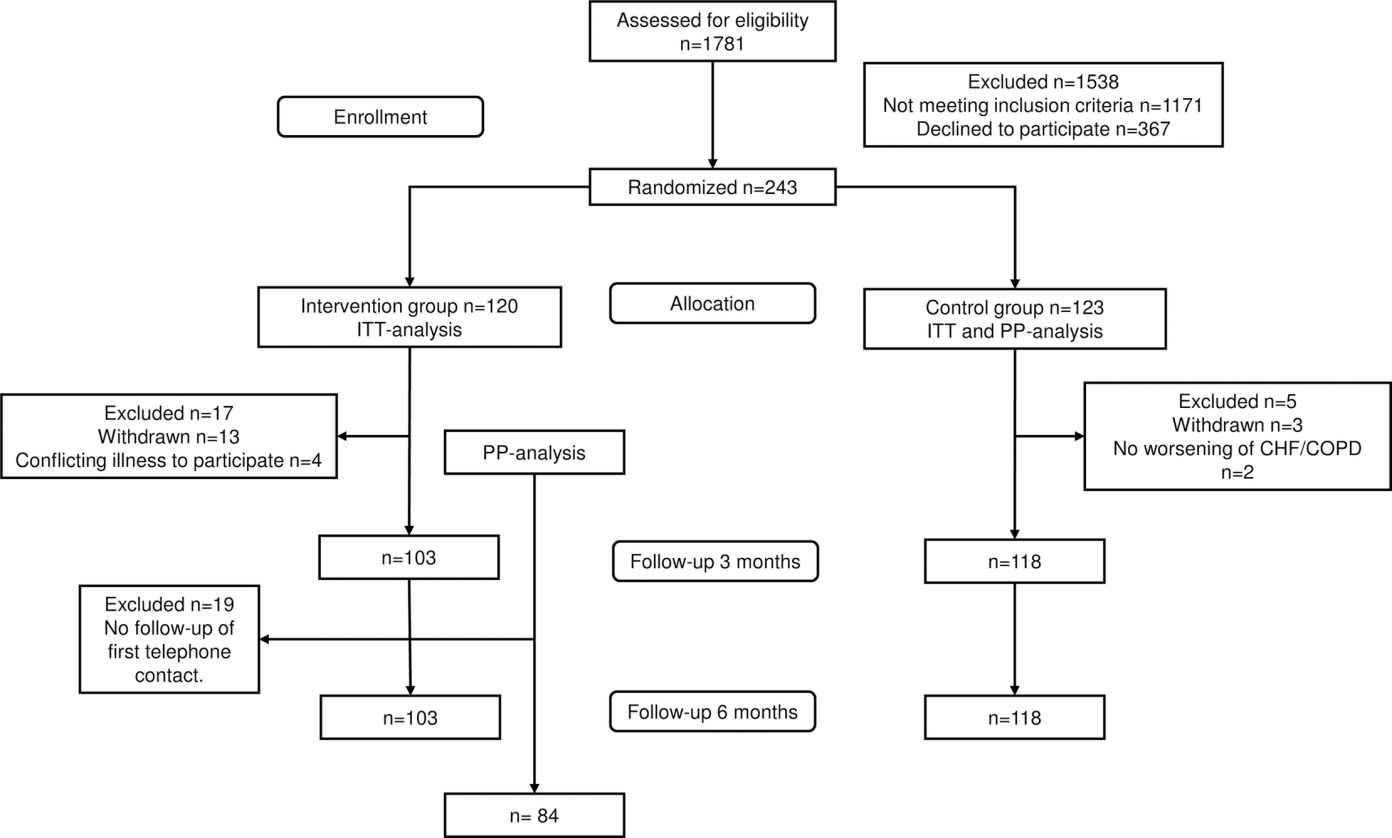
Trial profile.

Overall, 1781 patients were screened. Of these, 610 patients met eligibility criteria and 367 declined to participate; accordingly, 243 patients were randomly assigned to the two treatment arms (Fig 1). Thereafter, 6 patients were found to not meet inclusion criteria and were excluded and 16 patients withdrew consent leaving 118 patients in the control group and 103 patients in the intervention group. The mean age was 77.6 years and 54.3% were women. Baseline characteristics did not differ significantly between groups (Table 1). Four patients died before their first scheduled telephone contact. Telephone support was used on average 81.6 min per patient (median 70.0 min, range 0–378 min) during the six-month study period. In total, 332 telephone calls (median 3.0, range 0–10) were conducted out of which 309 were called by RNs and 23 by patients. Nineteen patients only used the telephone support once, without a follow-up contact, Therefore, these patients were censored in the PP analysis. Consequently, the PP analysis included 84 patients from the intervention group and all patients in the control group.

**Table 1:** Baseline characteristics.

	Control (n = 118)	Intervention (n = 103)
Age, years (mean(SD))	76.9(8.3)	78.3(9.5)
Female(%)	69(58.5)	51(49.5)
BMI (mean(SD))	26.7(6.4)	26.2(7.0)
General Self-Efficacy Score(mean(SD)	28.5(5.8)	28.1(6.5)
**Civil status(%)**		
Living alone	63(53.4)	62(60.2)
Married/partner	55(46.6)	41(39.8)
**Diagnose(%)**		
COPD	59(50.0)	50(48.5)
CHF	44(37.3)	44(42.7)
COPD and CHF	15(12.7)	9(8.7)
**Medical history(%)**		
Previous MI	26(22.0)	23(22.3)
Previous angina	23(19.5)	15(14.6)
Atrial fibrillation	45(38.1)	50(48.5)
Hypertension	61(51.7)	61(59.2)
CABG	12(10.2)	10(9.7)
Stroke	11(9.3)	15(14.7)
Diabetes	27(22.9)	29(28.2)
CRT	4(3.4)	1(1.0)
Pacemaker	13(11.0)	10(9.7)
Current or previous smoker (%)	86(73.5)	68(66.7)

BMI = body mass index; COPD = chronic obstructive pulmonary disease; CHF = chronic heart failure; MI = myocardial infarction; CABG = coronary artery bypass grafting; CRT = cardiac resynchronization therapy.

### 3.1 Effects

At six-month follow up no difference in the primary endpoint was found between the usual care and intervention groups (57.6%, n = 68 vs. 46.6%, n = 48; OR = 1.6, 95% CI: 0.9–2.7; P = 0.102) (Table 2). Splitting the composite score into each component separately in the ITT analysis showed that significantly more patients in the control group had deteriorated in self-efficacy (GSE scores ≥ 5 units) at the six months follow-up than in the intervention group (22.9%, n = 27 vs. 9.7%, n = 10; OR = 2.8, 95% CI: 1.3–6.0; P = 0.011). The same pattern was seen at three months (23.7%, n = 28 vs. 11.7%, n = 12; OR = 2.4, 95% CI: 1.1–4.9; P = 0.022). Improvement in GSE was significantly greater in favour of the intervention group at both three months (0.7 (mean) ± 5.8 (SD); n = 79 vs. -2.2 (mean) ± 6.1 (SD); n = 89; P = 0.010) and six months (0.9 (mean) ± 6.4 (SD); n = 69 vs. -2.0 (mean) ± 6.8 (SD); n = 85; P = 0.006). At six months there were no differences in clinical event rates: 49 events (14 deaths, 35 patients were re-admitted) in the control group and 41 events in the intervention group (9 deaths, 32 patients were re-admitted). In total, when including all events and not only the first event that occurred to a patient, there were 95 events in the control group (21deaths, 74 re-admissions) and 61 events in the intervention group (16 deaths, 45 re-admissions).

**Table 2:** Primary endpoint.

	Intervention	Control			
Intervention vs. control group (ITT analysis)	n = 103	n = 118			
Composite score			P-value 0.102*	OR 1.558	CI 95% 0.915–2.653
Deteriorated n(%)	48(46.6)	68(57.6)			
Unchanged n(%)	41(39.8)	42(35.6)			
Improved n(%)	14(13.6)	8(6.8)			
Intervention vs. control group (PP analysis)	n = 84	n = 118			
Composite score			P-value 0.039*	OR 1.813	CI 95% 1.030–3.193
Deteriorated n(%)	36(42.9)	68(57.6)			
Unchanged n(%)	36(42.9)	42(35.6)			
Improved n(%)	12(14.3)	8(6.8)			

*Table shows logistic regression of composite score at 6 months dichotomised into deteriorated vs. improved/unchanged as well as how many in each group that was deteriorated, unchanged or improved.

OR = odds ratio; CI = confidence interval.

In the PP analysis, there was a significant deterioration in the composite score in the control group compared with the intervention group (57.6%, n = 68 vs. 42.9%, n = 36; OR = 1.8, 95% CI 1.0–3.2; P = 0.039) at six months (Table 2). 49 events (14 deaths, 35 re-admissions) occurred in the intervention group vs. 29 events (3 deaths and 26 re-admissions) in the control group. The effect in the PP-analysis was also observed at three months (50.8%, n = 60 vs. 33.3%, n = 28; OR = 2.1, 95% CI 1.2–3.7; P = 0.014).

## 4. Discussion

In this RCT evaluating a person-centred telephone support in patients with COPD and/or CHF we found no significant between-group difference in the composite score consisting of general self-efficacy, rehospitalization and death; however, the PP analysis, including only those patients who met protocol specifications for fully implemented intervention (at least one follow-up of the first contact by telephone), showed a significant deterioration in the composite score in the control group at both three and six-month follow-up. Also at both follow-ups, significantly more patients in the control group showed a clinically important deterioration in self-efficacy (GSE change score ≥ five units) [12]. Moreover, GSE scores differed significantly between groups, where they decreased in the controls but increased in the intervention group. These results indicate that person-centred telephone support mitigates worsening general self-efficacy levels without increasing the risk of increased clinical events.

This study evaluated the effects of a person-centred intervention delivered solely by means of telephone support and our results indicate that PCC can be performed without requiring face-to-face consultations. Noteworthy is that the study was conducted in a vulnerable patient group comprising elderly patients with serious, progressive chronic conditions. With a mean age of 77.5 years, the patient group registered more than 150 disease-related events during the six-month study period and more than 40% (n = 90) had at least one diagnosis-related event, of which 25% were deaths (n = 23).

The effects in this study were driven by a change in general self-efficacy, i.e. belief in one´s abilities to manage various situations, which is a central mechanism to improve self-care and health [33]. The importance of evaluating effects on such an intermediate outcome, and not only ultimate outcomes such as health care utilization, has been stressed [34]. In the present study the decrease in self-efficacy shown in the control group might have been due to the condition being stigmatizing and “blame yourself” condition, whereas the intervention group was supported and strengthened in their abilities and self-respect. Previous studies using PCC in face-to-face interventions in several contexts, conditions and health care levels have been shown to reduce uncertainty about the illness [6] and length of hospital stay [5] in patients with CHF. Self-efficacy can be improved by engaging patients in problem-solving and developing their skills to manage their health problems and their particular challenges [33]. Still, factors contributing to building engagement are unknown [35] and patients with chronic conditions such as COPD are a particularly vulnerable and self-efficacy improvements are especially difficult to attain in such patients [34]. A similar pattern is seen in telehealth interventions where benefits have been reported in patients with CHF whereas they are rare in patients with COPD [26].

In a review synthesizing self-efficacy enhancing interventions in chronic conditions, self-efficacy was reported to be associated with improved concordance to medication, diet, physical activity, stress management and lower utilization of care after 1 year and health distress [36]. Moreover, studies in patients with cardiac disease have shown that self-efficacy is a predictor for several beneficial outcomes during recovery including healthier lifestyle [37], self-management behaviours, psychological well-being, health-related quality of life (HRQoL) [38] and attendance to cardiac rehabilitation programs [39].

In patients with COPD, higher self-efficacy levels are associated with lower levels of breathlessness, anxiety and depression [40] and it has also been shown to be a predictor of reduced psychosocial impact of COPD, improved physical activity levels and better HRQoL [34]. In contrast, self-management interventions for patients with COPD have showed mixed results to increase self-efficacy [34]. This may be due to the content of the interventions, which consisted mainly of informing patients about the disease, medications, smoking and exercise while few focused on self-monitoring and support. In our study we implemented a PCC approach that emphasizes the importance of identifying patients’ capabilities, resources, needs, preferences and barriers to self-management by listening carefully to their illness history.

Our PCC intervention also emphasized the importance of building a partnership between the patient and healthcare professionals based on ethical principles and mutual respect. Although it is difficult to pinpoint the most powerful active components to reduce the risk of a worsened self-efficacy, we suggest that the use of person-centred communication skills combined with a health plan were mediating factors to promote patient engagement and establish a partnership between the healthcare professionals and the patient. The intervention was performed at distance via telephone and required only a mean time of 80 minutes per patient during the six-month study period, suggesting that this intervention is minimally resource intensive.

## 5. Study limitations

Our study has limitations. Baseline variables such as NYHA class and stage of COPD are missing because they were not consistently reported in medical records. More than half of the eligible patients declined to participate. This may be due to a high morbidity and symptom burden which is reflected by high event rates among those who consented to participate. Six months is a relatively short period for follow-up and the effect of intervention could be diluted overtime so that the potential benefit for intervention group could disappear after 6 months. Even if PP analysis is common in interventional trials to analyze adverse effects [41], such analyses cannot replace ITT analysis.

## 6. Conclusions

In patients with CHF and/or COPD, with many presenting both conditions concomitantly, we found that person-centred telephone support reduced the risk of decreased self-efficacy without increasing the clinical events up to six months post discharge. Person-centred communication skills in combination with a health plan can be used to build a professional relationship between patients and healthcare providers, and given that the telephone-support was not particularly resource intensive it could be fairly easy to implement.

## Supporting information

S1 File Study protocol. (PDF)

S2 File CONSORT checklist. (PDF)
