# Supplementary material for: Effects of a person-centred telephone-support in patients with chronic obstructive pulmonary disease and/or chronic heart failure – A randomized controlled trial
Source: PLoS One. 2018 Aug 31;13(8):e0203031. doi: 10.1371/journal.pone.0203031 (PMC6118377; doi:10.1371/journal.pone.0203031)
Supplement: S1 File — (PDF) [file pone.0203031.s001.pdf]

**Care4ourselves – Person-centred care at distance**

# C4

| | |
|---|---|
| Principal Investigator | Inger Ekman, Professor, Director of University of Gothenburg Centre for Person-Centred Care |

# 1 Rationale and background information

Worldwide, health care systems are facing immense challenges: As life expectancy increases at unprecedented levels, an ever-growing aging population suffers from non-communicable, chronic disease (WHO, 2013). In order to address these problems with the limited financial and human resources available, new ways of delivering healthcare need to be explored. Tele-solutions (e.g. telehealth, telemonitoring, eHealth and mHealth services) are increasingly employed to enable the delivery of accessible, high-quality and cost-effective remote healthcare (WHO, 2011).

COPD and CHF are two long-term conditions, known for high re-hospitalisation rates and their severe impact on life expectancy and activities of daily living due to frequent symptoms of fatigue and dyspnoea that limit several aspects of their life, making patients dependent on others in their daily living (GOLD, 2013). Patients with CHF and COPD are mostly elderly and require hospital care frequently. Although pharmacological therapy has improved outcome markedly over the last 10-15 years, management programs are needed to optimize care (Holland et al., 2005). Being affected by a chronic disease with a high symptom burden is a challenge to living a fulfilled life. Support and training to enable successful self-management positively impacts on health behaviour, health status and health care utilization.

The World Health Organization and Institute of Medicine at the US National Academy of Sciences have identified person-centred care (PCC) as a core concept for the delivery of care in the chronically ill patient (Epping-Jordan, Pruitt, Bengoa, & Wagner, 2004; Organization., 2008). In PCC, the relationship between health care professional and patient is characterized by equality: professional and patient jointly develop a personal health plan using resources identified in each patient´s illness history but also by defining potential barriers (Ekman et al., 2011). GPCC research demonstrated the beneficial effect of PCC (shortened hospital stays) in a hospital environment (Ekman et al., 2012) as well as in relieving uncertainty of the condition and its treatment (Dudas et al., 2013).

C4 aims to deliver person-centered care (PCC) utilizing information and communication technology (ICT) for people with chronic heart failure (CHF) and / or chronic obstructive pulmonary disease (COPD).

Within this research project we will develop and test a telephone support service and ICT platform to provide information, training and professional support for patients. Patients will keep and manage their own health record ('Health Plan').

The goal is to enable patients to understand their disease and therapy better, to identify their own resources to enhance coping and living with their chronic illness by means of a dialogue and partnership between health carer and patient. This will be an advice-only service that does **not entail** changes in medical treatment plans or advice regarding medical decisions, investigations.

### *Aims of the project*

The goal of the two phases (Phase I and Phase II) of the research project C4' described in this plan is to translate the PCC principles into a tele context.

### *Project design*

This project is a complex intervention and as such features a multitude of influencing factors (May, Mair, Dowrick, & Finch, 2007) The study design was guided by the new Medical Research Council's (MRC) framework for complex interventions (Craig et al.,

2008). We opted for the revised version of the original MRC framework due to its iterative approach and increased focus on early phase piloting and development of complex intervention.

The two phases of the C4 project correspond with the three steps of *Development and Feasibility and Piloting* and *Evaluation* of the MRC framework (Craig, et al., 2008). The i*mplementation* of the intervention into a large context will be planned according to the results of this preliminary study in a follow-on protocol.
As the blue arrows in Figure 1 above indicate, *Development, Feasibility and Piloting*, and *Evaluation* are not distinct, stand alone phases in the MRC framework, but stages that may be revisited iteratively to allow for maximum flexibility and optimisation of the intervention and its delivery. The two phases of our project reflect this flexible and iterative approach as we embedded an RCT study into a number of qualitative subprojects. During phase I (April 2014 until October 2014), the preparation phase, we will develop the intervention and test the procedures. The development will be guided by qualitative interview studies aimed at exploring patients support needs and the nurse-patient relationship in telecare. The knowledge gained in this phase will inform the development of the actual intervention.

During phase II (October 2014 until December 2015), we will test and evaluate the procedures with in an RCT supplemented by qualitative studies. The quantitative study, an RCT will be conducted to research projects to evaluate our primary endpoints (patient and carer outcomes and economic evaluation of the intervention). While the quantitative assessment is essential to demonstrate the effect of the intervention on health care utilization and patient outcomes, we decided to supplement this study with various qualitative subprojects. This will enable us to obtain feedback on the impact of the intervention on patients and their informal carers, but also the effect on nurses who deliver a PCC based intervention.

The two phases and their subprojects will be described in detail in the respective sections of this study plan.

Figure 1 gives an overview of the two main phases of the project.

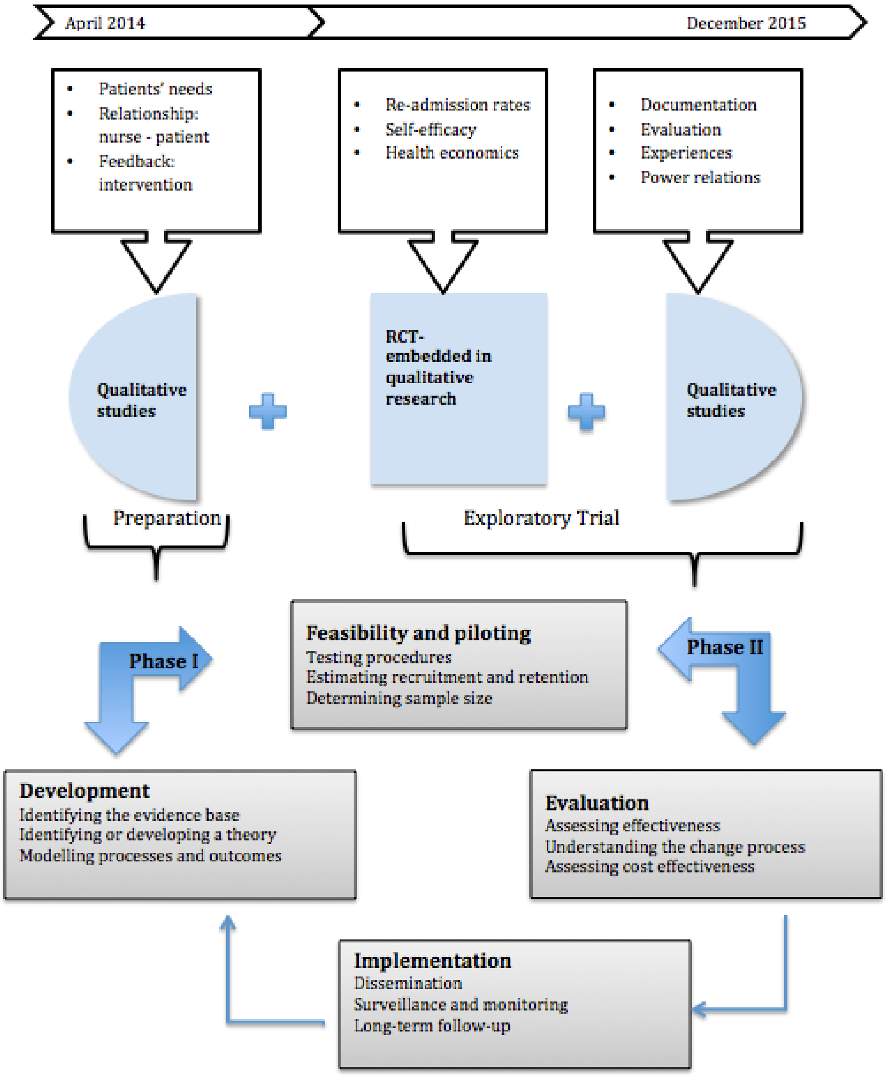


**Figure 1: Phases I and II of C4 within the MRC framework (Craig, et al., 2008)**

# 2 Project Plan phase I – Preparation (April 2014 – October 2014)

During phase I, we will explore patient needs and nurses perspectives on the delivery of remote health-care through qualitative interview studies. The results obtained in both studies will inform the design and delivery of the patient intervention.

## 2.1 Interview study exploring patients' support needs

| | |
|---|---|
| Background | Patients suffering from chronic obstructive pulmonary disease (COPD) and/or chronic heart failure (CHF) experience a host of restrictions in daily life. Apart from physical restriction, they have to cope with anxiety, fear of living as well as fear of dying, fear of the future and a reluctance to engage with the feelings triggered by the disease (Strang, Ekberg-Jansson, & Henoch, 2013) |
| Aim | To investigate the need for PCC in patients suffering from COPD and/or CHF. |
| Research Question | What are the care needs of patients suffering from COPD and/or CHF? |
| Method | Individual interviews, semi structured. |
| Sample & sampling | A convenience sample of 15-20 (depending on point of data saturation) volunteers affected by COPD and/or CHF, recruited via a facebook page for peer-support (Lugna Lungor), patient organizations ('Riksförbundet HjärtLung') and a flyer (Verdandi) between April-August 2014. |
| Data collection and analysis | Individual interviews with patients suffering from COPD and/ or CHF, to be transcribed and content analysed (Graneheim & Lundman, 2004; Krippendorff, 2004). |
| Ethical considerations | The respective ethics committee was contacted and presented with the study proposal. The committee raised no concerns and did not require a full application for ethical approval. Written informed consent (Appendix A) was obtained from all participants. Personal identifiable data will be coded in the transcription. |
| Investigators | Lilas Ali, Birgit Heckemann, Inger Ekman |

## 2.2 Interview study focussing on the experience of nurses working in telecare

| | |
|---|---|
| Background | Registered nurses (RNs) who deliver care from call-centres have no face-to-face encounters with patients. While some studies have explored the role of the RNs working in tele-care to some extent (Chang, Lee, & Mills, 2013; Doolittle, 2001), in-depth studies exploring the tele care relationship between patient and nurse, as well as the challenges and opportunities which are associated with tele- care delivery are lacking. |
| Aim | To explore the possibility of integrating PCC concepts into a telehealth context. |
| Research Question | How do nurses perceive their call center environment to differ from a traditional hospital ward or clinic environment? |

| | |
|---|---|
| | How does the remote care delivery and their virtual presence alter the patient – nurse relationship?<br>How does the remote care delivery affect RN's professional identity and role?<br>Where are the barriers and opportunities to the delivery of person-centred care in a telecare context? |
| Method | Focus group interviews, semi-structured. |
| Sample & sampling | 4 focus groups with a total of 21 RNs working in a German telehealth centre.<br>Data analysis: Qualitative content analysis (Graneheim & Lundman, 2004; Krippendorff, 2004) |
| Data collection and analysis | Semi-structured focus groups interview study with RN's working in a telehealth centre in Southern Germany. The centre provides care for more than 10000 chronically ill patients across the country. |
| Ethical considerations | The protocol for this study was reviewed by the respective German ethical committee: Ärztekammer Bayern Ethikkommission, Bayerische Landesärztekammer in Munich in May 2014 and declared as ethically safe. The committee raised no concerns and did not require a full application for ethical approval. Written informed consent (Appendix B) was obtained from all participants. Personal identifiable data will be coded in the transcription. |
| Investigators | Birgit Heckemann, Lilas Ali, Axel Wolf, Inger Ekman |

## 2.3 Preparation of nurses for delivery of tele-support

To prepare nurses to deliver PCC care through a training program based on the principles for Person-centred Care as outlined by Ekman et al. (2011).

**Proposed contents of nurse training (as identified during workshop on 18/19 August 2014**) Nurses will develop and practice their skills under the guidance of experienced mentors (I. Lindström and A. Fors)

**The training will include the following aspects, it will be developed based on the feedback from the nurses.**

### Medical knowledge about COPD and CHF

Areas that will need to be addressed in the theoretical training on facts about COPD and CHF as identified in the workshop are:

- COPD and CHF as a disease, symptoms, causes, disease progression, treatment, etc.
- Medicines, their use, side effects, administration
- Medical technical products (e.g. inhalers) and their use
- Frequent patient questions about disease and its management
- Knowledge about co-morbidities that affect the patients, such as diabetes

### Living with COPD/ CHF

Knowledge about resources for the patient and how to access these.

- Formal support structures that exist within the community (mobile team, COPD teaching group, access to specialist nurses and doctors etc.)

- Informal support structures: patient groups, networks for patients and informal carers
- Advice on daily living: Organize training sessions for nurses with
    - ‘Occupational therapist’
    - ‘Physiotherapist’
    - ‘Dietician’

## Eliciting the patient narrative: Communication techniques

The patient narrative is the key to establishing a person centred partnership between patient and nurse, to identify the patient’s goals and resources, and to document these in a mutually agreed Health plan.

The communication training will need to comprise practical and theoretical elements.

Theoretical elements:

- Motivational interviewing
- Person-centred Communication as per gPCC (Fors, 2014)
- Enabling and sense making model of communication (Ohlen, Wallengren Gustafsson, & Friberg, 2013)

Practical elements:

- Simulation of telephone conversations with patient representatives and nurses
- Recording of communication, reflection and analysis on person-centredness of conversation with mentors and peers

## Coaching and Counselling skills

In order to enable patients

- To become more equal in their encounters with other health professionals such as their cardiologist, pulmonologist or General Practitioner, patients need to feel confident and possess relevant knowledge. Coaching and counselling to take a more active in decision-making processes in clinical will be part of the tele-support service.
- To better learn to manage the anxiety and fear associated with having to live with the symptoms of a chronic disease (e.g. guiding patients towards resources such as mindfulness training)

Theoretical elements: Introduction into coaching and counselling

Practical elements:

- Simulation of telephone conversations with patient representatives and nurses
- Recording of communication, reflection and analysis on person-centredness of conversation with mentors and fellow nurses

## Building an equal partnership

Theoretical elements: Exploring the philosophical underpinnings of PCC.

Practical elements: Personal reflection in learner’s diary, informal reflection within research group

## Documentation and personal Health plan

Theoretical elements: Input sessions on documentation and personal Health plan as per gPCC principles.

Practical elements: Reflection and practical utilization in peer discussion and teaching sessions.

# 3 Project plan phase II- Evaluation- (October 2014 – December 2015)

*A Person- centred Care-based tele-support for patients (PCC-PTS)*

This intervention, based on the definition of PCC as per Ekman et al (2011), will rest on four 'pillars': *Personal health plan, Educational package, Coaching & counselling, Symptoms & signs.*
Below is a description of the overall project, followed by brief descriptions of the individual subprojects.

## 3.1 Aim

Within the C4 program, we aim to deliver support to patients with chronic obstructive pulmonary disease (COPD) and chronic heart failure (CHF) and their informal care givers to live better and cope more effectively with the disease burden associated with COPD and/ or CHF.

## 3.2 Scope

The goal is to enable patients and their informal carers to understand COPD and/or CHF and the therapy better, to identify their own resources to enhance coping and living with their chronic illness by means of a dialogue and partnership between health carer and patient. This will be an advice-only service that does **not entail** changes in medical treatment plans or advice regarding medical decisions, investigations

## 3.3 Hypothesis

Inclusion of the principles of PCC in a tele-partnership system for patients with CHF and COPD will reduce the need for medical care (primary care and hospital re-admission) amongst these patients by improving self-management, self-efficacy and collaboration in the process of care.

## 3.4 Endpoints

### 3.4.1 Primary endpoints

The study will be conducted under the assumption the PCC relationships can easily be created at distance. The objective is to elaborate if PCC delivered through a tele-support service aimed at patients suffering from CHF and COPD will change the primary outcome measure: self-efficacy.

**Primary outcome measure: Self-efficacy**

| | |
|---|---|
| Improved: | Increase in Self-efficacy> 5 units at 6 months and has not been re-admitted to hospital |
| Worsened: | Death or re-admitted to hospital within 6 months or a decrease in self-efficacy> 5 units |
| Unchanged: | Self-efficacy has neither improved or deteriorated |

### 3.4.2 Secondary endpoints

a. Number of readmissions
b. Change in Self-Efficacy
c. Health care utilization, measured as the number of re-admissions, and unscheduled outpatient visitsduce unplanned visits to hospital and/or vårdcentral due to symptoms of COPD and CHF

## 3.5 Study design

As outlined in section the overall study design is an experimental embedded research design (see also figure 1). In phase II of this project, we will run a randomized Controlled pilot study over a six months period. The intervention group will receive the PCC-PTS programme, the control group will receive usual care. This study is embedded in qualitative studies to obtain a better understanding of the mechanisms that affect the outcomes of the intervention.

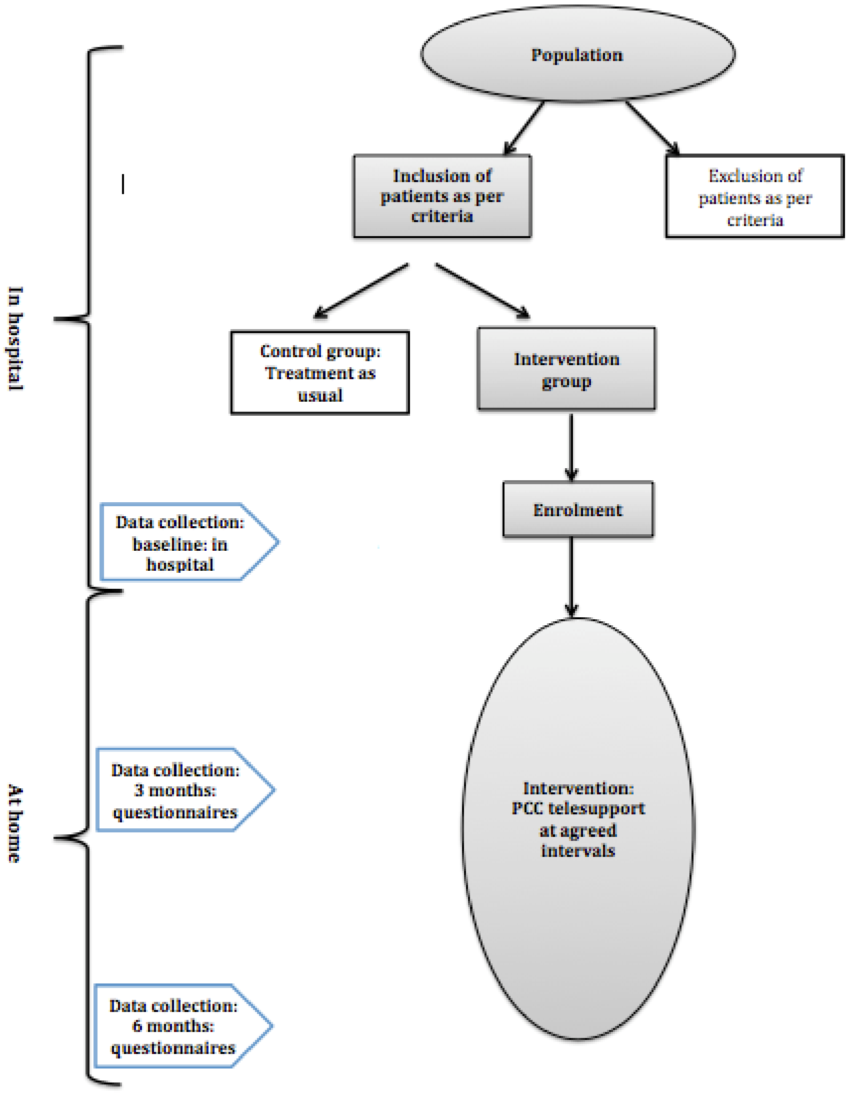

Figure 2: Flowchart: RCT: Intervention and study design

## 3.6 Population and sample

Adult patients who have been hospitalized due to worsening CHF and/or COPD in one of Gothenburg's major hospitals (Sahlgrenska, Östra Sjukhuset) or to Skellefteå hospital.

### 3.6.1 Enrolment

Patients will be enrolled to the program during their hospital stay, either from A&E or on the ward once their condition has stabilized sufficiently.
Specially trained RNs will screen and inform patients about the aims of the study, as well as its potential benefits for patients (see screening from in Appendix A). Patients will need a smartphone to participate in the study. Patients who do not own a smartphone will be provided with one for the duration of their participation in the programme.

### 3.6.2 Selection: control vs. intervention

Patients admitted to hospital for worsening COPD and/or CHF will be recruited by the research nurses employed in this project from Emergency or on the ward once their condition has stabilized sufficiently.
Patients will be screened against inclusion and exclusion criteria for eligibility to participate in the study. Written informed consent will be obtained. Patients will be randomized into either the control or the intervention arm 1:1 . The control group will receive usual care. The intervention group will receive usual care plus a PCC nurse-led intervention. Randomization will be done through computer generated lists and stored in sealed envelopes.

### 3.6.3 Inclusion criteria

- History of confirmed diagnosis of COPD and /or CHF
- Admission to hospital for relapse /worsening symptoms of COPD and/or CHF
- Age: $\geq 50$
- National registration within the Västra Götaland Region (VGR)
- Owning a phone and current subscription to a telephone service provider

### 3.6.4 Exclusion criteria

Severe hearing impairment that prevents patient from using a telephone or other communication device
Patient has no registered address
Any severe disease with an expected survival < 12 months
Cognitive impairment (SPMSQ score >6)
Ongoing documented diagnosis of alcohol or drug abuse
Other disease that can interfere with follow-up (e.g. severe depression, other severe mental illness)
Patient participating in another randomized study

### 3.6.5 Number of patients – Power analysis

To achieve 80 % power with an alpha-error of 0.05 for an increase in the number of patients with improved self-efficacy>5 units from 20% to 40%, the number of participants in each group (comparison and intervention) needs to comprise 91 patients. We aim to include 110 patients in each group to have some margin for withdrawals/ dropouts

## 3.7 The intervention

### 3.7.1 The patient programme: Contents or 'Building blocks' of the intervention

The intervention consists of a communication and on-going dialogues between patients, their informal carers and tele-support nurses. The communication with the patient will be based on the principles of person-centred communication (Listening, open questions, reflective listening and summarising of narrative) (Fors, 2014).

The intervention comprises four main ‘building blocks’.

‘Building blocks’ of the intervention:

- **Coaching, motivation and empowerment** of the patient and informal carer: Patients suffering from a chronic disease often experience anxiety and depression. These may worsen disease symptoms and hamper effective self-management (DeJean, Giacomini, Vanstone, & Brundisini, 2013). Coaching and counselling towards self-management of patients (and their informal caregivers) will be part of the programme.
- **Pedagogical conversations:** Discussing the disease, medical treatment, and its management in daily life as per patient’s needs: Patients suffering from COPD and/or CHF do not receive education about their disease that is tailored to their needs, so they often have minimal knowledge about their condition. The program aims to fill knowledge gaps by providing adequate information as per the patient’s (and informal care givers’) needs and abilities.
- **Monitoring of signs and symptoms** via a smartphone application (Symgo)
- **A personal Health Plan:** A regularly updated record of a mutually agreed health plan based on the patient’s illness narrative. The personal Health Plan is a document for tracking care processes, it is maintained and held by the patient. This personal plan will be re-evaluated on a frequent basis, on every appointment with the tele-nurse. The health plan will contain patients’ aims and goals for living with and management of their disease.

### 3.7.2 Delivery of the intervention

The delivery of the intervention will commence during the patient’s hospital stay. In an initial face-to-face meeting between patient and nurse, the patient’s illness narrative will be elicited and the mutually agreed personal Health Plan will be formulated in partnership with the patient. The plan comprises a written record of the patient’s personal goals and aims. Aims and goals may relate to

1. Regular physical activity
2. Regular social activity
3. Symptom management
4. Increasing wellbeing
5. Or other personal aims or goals as identified by the patient

As per patient preference, the health plan will be either in electronic or paper format. The patient keeps the Health Plan, yet might give read-only access to other health care providers such as hospitals or primary care centres.

## The Health Plan

- THE Core document for tracking patients' aims and goals in the care relationship
- Based and formulated on the condensed patient narrative
- Will not include medical information included in other patient records
- Mutually agreement of goals and aims between patient and nurse, identification of patient's resources and support needs.
- Also contains: follow-up actions, steps to be taken towards achieving the goals, planning and documentation of care processes in the community: e.g. primary care centre, social welfare centre, appointment times and dates
- Held and maintained by the patient who may give read-only access to care providers (e.g. Hospital, primary care centre)
- Regularly revised and updated during each patient-nurse contact: The nurse will update the plan and share it with the patient for his or her comments.
- May be in paper or electronic format, depending on patient preference

**Figure 2: The Health Plan**

#### *3.7.2.1 During the hospital stay: Initiating the contact*

The nurse will visit the patient to formulate the patient's personal Health Plan in partnership. Based on the initial dialogue with the patient, the nurse will draft the HP and share this draft with the patient for comments and approval. Once approved by the patient, the HP will be uploaded onto an online personal health diary that allows the patient to access the HP. The patient can also grant read-only access to the HP to care providers such as the hospital, outpatient clinics or primary care centres. A copy of the HP will also added to the patient's medical journal as a research note.

#### *3.7.2.2 At discharge*

On discharge, the nurse and patient will meet again to revisit the HP and to schedule the first telephone support call. If needed, the patient may also contact the nurse prior to the scheduled telephone support call.
If the PCC health plan is updated, the PCC nurse will send the updated version both to the patients' online health diary as well as the medical journal as a research note.

#### *3.7.2.3 At home*

After discharge from hospital, the nurse will contact the patient at mutually agreed intervals to revisit personal goals, to educate, motivate and coach the patient, as well as to discuss symptom management. During each telephone call, the HP will be updated by the nurse and then shared with the patients for comments and approval. New appointment times will be scheduled at each telephone support call. Patients informal carers will be involved.
Supplementary support: The service will be set up in collaboration with representatives from Swedish patient support groups (Riksförbundet HjärtLung), so patients will also be offered access to a network of support initiatives organised by patients. Each patient will be looked after by a 'pool' of two to three nurses to ensure that patients benefit from the wide range of professional experience. If patients are being re-admitted for

worsening condition during the intervention period, the support will continue once they have been discharged from the respective hospital episode.

### 3.7.3 Technical features

During the hospital stay, the patient can familiarize her/himself with the personal support record, which consists of

- The Health Plan
- A personal diary
- An instrument to track trends in signs and symptoms over time (e.g. breathlessness, fatigue)

The patient will receive an individual user name and access to the diary will be password protected. The diary may be accessed via smartphone, tablet or computer. All data will be stored, managed and be made accessible in line with PuL (Personuppgiftslagen) legislation and with Gothenburg University's guidelines on data protection.

## 3.8 Baseline data

**Demographics:** Sex, age (year), education, employment, socio-economics, marital status.
**Characteristics:** Height, weight, waist and hip.
**History**: Smoking, Co- morbidity; Chronic obstructive pulmonary disease, Chronic heart failure, Stroke, Atrial fibrillation/flutter, Diabetes, Hypertension and Cancer.
COPD – number of admission before current indexed admission

### 3.8.1 Assessment

**Self assessment** at baseline and follow-up (3 and 6 months)
Patients

- Health related Quality of Life - EQ-5D (Brooks, 1996; Wittrup-Jensen, Lauridsen, Gudex, & Pedersen, 2009)
- General Self- Efficacy scale (Schwarzer & Jerusalem, 1995),
- Cardiac-Self Efficacy Scale (Fors, Ulin, Cliffordson, Ekman, & Brink, 2014),
- Beliefs about Medicines Questionnaire BMQ, specific (Horne, Weinman, & Hankins, 1999)
- Shortness of Breath in Heart Failure (SOB-HF) (Ekman, Granger, Swedberg, Stenlund, & Boman, 2011)
- COPD Assessment Test (CAT) (GlaxoSmithKline, 2009)
- Fatigue Impact Scale (FIS) (Flensner, Ek, & Soderhamn, 2005)
- Hospital anxiety and depression scale (HADS) (GL Assessments)

Carers:

- Cope Index

**Health Service utilization**

- Readmission rates to hospital for worsening COPD and/or CHF
- Visits to vårdcentral/hospital *to be updated*.

## 3.9 Statistical methods and analysis

The statistical analyses will have a significance level of 5 % and, when relevant, 95 % confidence intervals will be reported. Mean values will be compared using t-tests and non-parametric tests will be used for analysis of nominal and ordinal data. Logistic regression will be used to study the co-variation of descriptive variables in relation to the results.
Regarding descriptive statistics, absolute numbers and percentages will be calculated for nominal variables; means and standard deviations will be computed for normally distributed continuous variables; and medians and quartiles will be calculated for non-normally distributed variables.

The primary outcomes in the intervention and control groups will be compared by ….

All analyses will have a significance level of 0,05 %, and 2-sided tests will be used.

## 3.10 Coordination of the study

The study will be run and coordinated by the Centre for Person-centred Care (GPCC), Gothenburg University.

## 3.11 Management of data and statistics

All data will be stored, managed and be made accessible in line with PuL (Personuppgiftslagen) legislation and with Gothenburg University's guidelines on data protection.

## 3.12 Ethical considerations

| | |
|---|---|
| The study | This study will be subject to review by the regional ethics committee in Gothenburg. |
| The investigators | The overall responsibility for the ethical conduct of this study rests with the PI, Inger Ekman. |
| The intervention | The intervention consists of a conversation between patient and tele-nurse, it is as such of a purely therapeutic nature and we expect patients to benefit from the participation in the study and foresee no negative side effects.<br>The participants: All participants in this study will be asked to provide their written informed consent before taking part in the study. |
| The participants | Written informed consent will be obtained from all participants included in the study. Participants may withdraw their consent to take part in the study at any point in time without any consequences. |

## 3.13 Milestones

This study is scheduled to run between November 2014 and October 2015. The figure below outlines the milestones in the process.

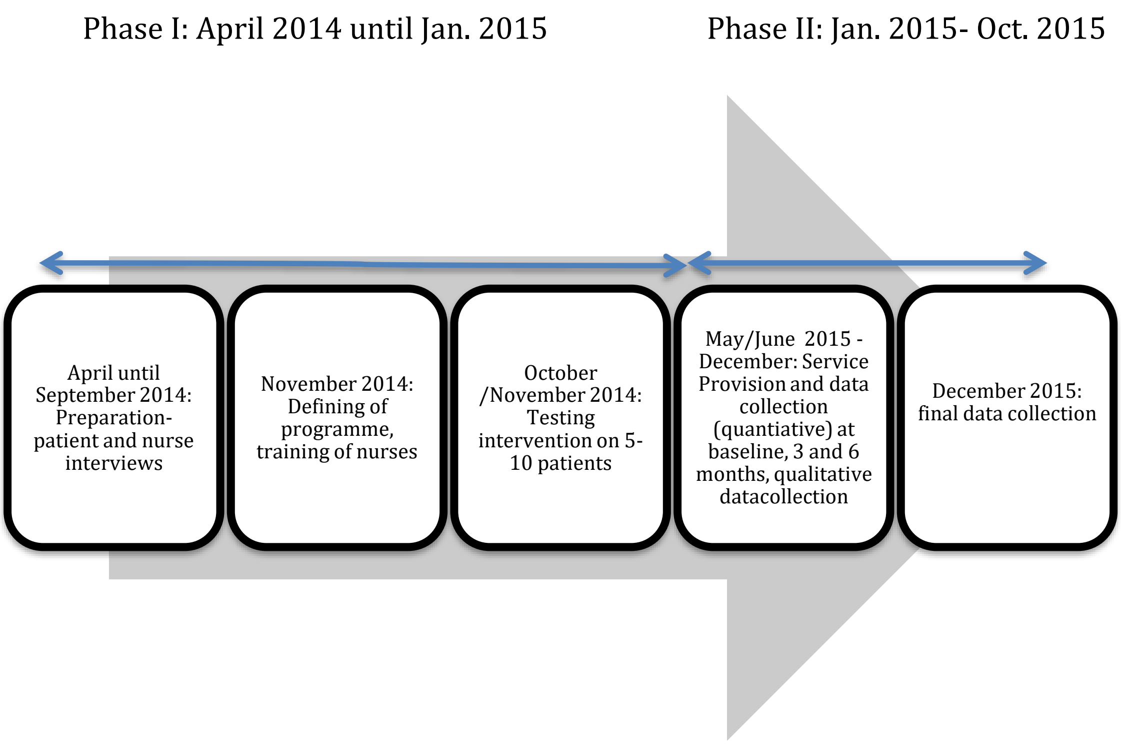

### *3.13.1.1 References:*


Brooks, R. (1996). EuroQol: the current state of play. *Health Policy, 37*(1), 53-72.

Chang, C. P., Lee, T. T., & Mills, M. E. (2013). Telecare for the elderly--community nurses' experiences in Taiwan. *Comput Inform Nurs, 31*(1), 29-35.

Craig, P., Dieppe, P., Macintyre, S., Michie, S., Nazareth, I., & Petticrew, M. (2008). *Developing and evaluating complex interventions: new guidance*: Medical Research Council (MRC).

DeJean, D., Giacomini, M., Vanstone, M., & Brundisini, F. (2013). Patient experiences of depression and anxiety with chronic disease: a systematic review and qualitative meta-synthesis. *Ont Health Technol Assess Ser, 13*(16), 1-33.

Doolittle, G. C. (2001). Telemedicine in Kansas: the successes and the challenges. *J Telemed Telecare, 7 Suppl 2*, 43-46.

Dudas, K., Olsson, L. E., Wolf, A., Swedberg, K., Taft, C., Schaufelberger, M., et al. (2013). Uncertainty in illness among patients with chronic heart failure is less in person-centred care than in usual care. *Eur J Cardiovasc Nurs, 12*(6), 521-528.

Ekman, I., Granger, B., Swedberg, K., Stenlund, H., & Boman, K. (2011). Measuring shortness of breath in heart failure (SOB-HF): development and validation of a new dyspnoea assessment tool. *European Journal of Heart Failure, 13*, 838 - 845.

Ekman, I., Swedberg, K., Taft, C., Lindseth, A., Norberg, A., Brink, E., et al. (2011). Person-centered care — Ready for prime time. *European Journal of  Cardiovascular Nursing, 10*(4), 248-251.

Ekman, I., Wolf, A., Olsson, L. E., Taft, C., Dudas, K., Schaufelberger, M., et al. (2012). Effects of person-centred care in patients with chronic heart failure: the PCC-HF study. *Eur Heart J, 33*(9), 1112-1119.

Epping-Jordan, J. E., Pruitt, S. D., Bengoa, R., & Wagner, E. H. (2004). Improving the quality of health care for chronic conditions. *Qual Saf Health Care, 13*(4), 299-305.

Flensner, G., Ek, A. C., & Soderhamn, O. (2005). Reliability and validity of the Swedish version of the Fatigue Impact Scale (FIS). *Scand J Occup Ther, 12*(4), 170-180.

Fors, A. (2014). Personcentrerade samtal inom hälsopromotivt arbete. In I. Ekman (Ed.), *Personcentrering inom hälso- och sjukvård. Fran filosofi till praktik* (pp. 226-243). Stockholm, Sweden: Liber AB.

Fors, A., Ulin, K., Cliffordson, C., Ekman, I., & Brink, E. (2014). The Cardiac Self-Efficacy Scale, a useful tool with potential to evaluate person-centred care. *Eur J Cardiovasc Nurs*.

GL Assessments. The Hospital Anxiety and Depression Scale. from http://www.gl-assessment.co.uk

GlaxoSmithKline. (2009). COPD Assessment Test (CAT). online: GSK, 980 Great West Road, Brentford, Middlesex. TW8 9GS, United Kingdom.

GOLD. (2013). Global strategy for the diagnosis, managment, and prevention of chronic obstructive pulmonary disease. online: Global Initiative for Chronic Obstructive Lung Disease.

Graneheim, U. H., & Lundman, B. (2004). Qualitative content analysis in nursing research: concepts, procedures and measures to achieve trustworthiness. *Nurse Education Today, 24*(2), 105-112.

Holland, R., Battersby, J., Harvey, I., Lenaghan, E., Smith, J., & Hay, L. (2005). Systematic review of multidisciplinary interventions in heart failure. *Heart, 91*(7), 899-906.

Horne, R., Weinman, J., & Hankins, M. (1999). The Beliefs about Medicines Questionnaire: The development and evaluation of a new method for assessing the cognitive represenation of medication. *Psychology and Health, 14*, 1-24.

Krippendorff, K. (2004). *Content Analysis: An introduction to its methodology*: Sage Publications, Inc.

May, C., Mair, F., Dowrick, C., & Finch, T. (2007). Process evaluation for complex interventions in primary care: understanding trials using the normalization process model. *BMC family practice, 8*, 42.

Ohlen, J., Wallengren Gustafsson, C., & Friberg, F. (2013). Making sense of receiving palliative treatment: its significance to palliative cancer care communication and information provision. *Cancer Nurs, 36*(4), 265-273.

Organization., W. H. (2008). *Caring for people with chronic conditions*: World Health Organization.

Schwarzer, R., & Jerusalem, M. (1995). Generalized Self-Efficacy scale. In J. Weinman, S. Wright & M. Johnston (Eds.), *Measures in health psychology: A user's portfolio. Causal and control beliefs* (pp. 35-37). Windsor, UK: NFER-NELSON.

Strang, S., Ekberg-Jansson, A., & Henoch, I. (2013). Experience of anxiety among patients with severe COPD - A qualitative, in-depth interview study. *Palliat Support Care*, 1-8.

WHO. (2011). mHealth: New horizons for health through mobile technologies: second global survey on eHealth. Geneva, Switzerland: World Health Organization.

WHO. (2013). World health report 2013: Research for universal health coverage: World Health Organization, Geneva, Switzerland.

Wittrup-Jensen, K. U., Lauridsen, J., Gudex, C., & Pedersen, K. M. (2009). Generation of a Danish TTO value set for EQ-5D health states. *Scand J Public Health, 37*(5), 459-466.
